# Activation of JNK Contributes to Evodiamine-Induced Apoptosis and G_2_/M Arrest in Human Colorectal Carcinoma Cells: A Structure-Activity Study of Evodiamine

**DOI:** 10.1371/journal.pone.0099729

**Published:** 2014-06-24

**Authors:** Chih-Chiang Chien, Ming-Shun Wu, Shing-Chuan Shen, Ching-Huai Ko, Chih-Hung Chen, Ling-Ling Yang, Yen-Chou Chen

**Affiliations:** 1 Department of Nephrology, Chi-Mei Medical Center, Tainan, Taiwan; 2 Department of Food Nutrition, Chung Hwa University of Medical Technology, Tainan, Taiwan; 3 Division of Gastroenterology, Department of Internal Medicine, Wan Fang Hospital, Taipei Medical University, Taipei, Taiwan; 4 Graduate Institute of Medical Sciences, College of Medicine, Taipei Medical University, Taipei, Taiwan; 5 Strategic Business and Innovation Technology Development Division, and Biomedical Technology and Device Research Labs, Industrial Technology Research Institute, Hsinchu, Taiwan; 6 College of LOHAS, Fo Guang University, Yilan, Taiwan; 7 Cancer Research Center and Orthopedics Research Center, Taipei Medical University Hospital, Taipei, Taiwan; Tel-Aviv University, Israel

## Abstract

Evodiamine (EVO; 8,13,13b,14-tetrahydro-14-methylindolo[2′3′-3,4]pyrido[2,1-b]quinazolin-5-[7H]-one derived from the traditional herbal medicine *Evodia rutaecarpa* was reported to possess anticancer activity; however, the anticancer mechanism is still unclear. In this study, we investigated the anticancer effects of EVO on human colon COLO205 and HT-29 cells and their potential mechanisms. MTT and lactate dehydrogenase (LDH) release assays showed that the viability of COLOL205 and HT-29 cells was inhibited by EVO at various concentrations in accordance with increases in the percentage of apoptotic cells and cleavage of caspase-3 and poly(ADP ribose) polymerase (PARP) proteins. Disruption of the mitochondrial membrane potential by EVO was accompanied by increased Bax, caspase-9 protein cleavage, and cytochrome (Cyt) c protein translocation in COLO205 and HT-29 cells. Application of the antioxidant N-acetyl-L-cysteine (NAC) inhibited H_2_O_2_-induced reactive oxygen species (ROS) production and apoptosis, but did not affect EVO-induced apoptosis of COLO205 or HT-29 cells. Significant increases in the G_2_/M ratio and cyclinB1/cdc25c protein expression by EVO were respectively identified in colon carcinoma cells via a flow cytometric analysis and Western blotting. Induction of extracellular signal-regulated kinase (ERK) and c-Jun N-terminal kinase (JNK) protein phosphorylation was detected in EVO-treated cells, and the JNK inhibitor, SP600125, but not the ERK inhibitor, U0126, inhibited EVO-induced phosphorylated JNK protein expression, apoptosis, and G_2_/M arrest of colon carcinoma cells. Data of the structure-activity analysis showed that EVO-related chemicals containing an alkyl group at position 14 were able to induce apoptosis, G_2_/M arrest associated with increased DNA ladder formation, cleavage of caspase-3 and PARP, and elevated cycB1 and cdc25c protein expressions in COLO205 and HT-29 cells. Evidence supporting JNK activation leading to EVO-induced apoptosis and G_2_/M arrest in colon carcinoma cells is provided, and alkylation at position 14 of EVO is a critical substitution for treatment of colonic cancer.

## Introduction

Colorectal cancer (CRC) is the second leading diagnosed cancer with high mortality, and remains a significant global health problem [1], [2]. Many therapeutic strategies such as surgery and chemotherapy are used to treat CRC; however, there are troublesome side effects with chemotherapy, and surgical treatment is associated with high mortality and local recurrence [3], [4]. Natural products have served as a leading source of drug development for centuries, and many of the new antitumor drugs such as taxol and cisplatin are natural products or derived from natural products [5], [6]. Evodiamine (EVO) is a natural chemical isolated from *Evodia rutaecarpa*, and several biological effects of EVO including antitumor, antinociceptive, and vasorelaxant properties were reported [7], [8]. EVO showed an inhibitory effect on tumor cell migration in vitro, and induced cell death in several cell types, but had little effect on normal human peripheral blood mononuclear cells [9]. Ogasawara et al. (2004) indicated the inhibitory effects of EVO against the invasion and lung metastasis of colon carcinoma cells [10]. In addition to anti-tumor effect, EVO may inhibit insulin-Stimulated mTOR-S6K activation in adipocytes and improves glucose tolerance in Obese/Diabetic Mice [11]. These results reveals the beneficial effects of EVO, however the mechanism underlying its antitumor activities and the structure-activity relationship of EVO are still poorly defined.

Recent studies suggested that eradication of cancer cells by anticancer agents was mediated by induction of apoptosis of those cells [12]–[14]. There are several apoptotic pathways in cells in response to apoptotic stimuli, and induction of apoptosis by chemotherapeutic agents mostly occurs through mitochondrial apoptotic pathways [15], [16]. The release of mitochondrial apoptotic proteins such as cytochrome (Cyt) c initiates caspase activation, and Cyt c release leads to activation of caspase-9, which in turn activates effector caspases such as caspase-3 causing caspase-dependent DNA fragmentation, a characteristic of apoptosis. Members of the Bcl-2 family proteins with either proapoptotic (e.g., Bax, and Bak) or antiapoptotic (e.g., Bcl-2, and Bcl-xL) functions regulate the mitochondrial membrane permeability (MMP) in apoptosis, and decreases in antiapoptotic and increases in proapoptotic Bcl-2 family proteins were observed during apoptosis of cancer cells under chemical stimulation. Previous papers indicated that the subtle balance of the Bcl-2/Bax complex led to an anti- or proapoptotic effect, and the overexpression of Bax may induce loss of the MMP that initiates apoptosis progression [17], [18]. It was indicated that disruption of the MMP via disturbing the Bcl-2/Bax balance leading to activation of caspases-9 and -3 plays an important role in apoptosis induced by chemotherapeutic agents. Reactive oxygen species (ROS) are mediators of apoptosis induction, and a number of studies showed that increased ROS production can cause cellular apoptosis via a mitochondrion-dependent pathway [19]. EVO was shown to induce apoptosis in various cancer cells; however, the mechanisms and roles of ROS in EVO-induced apoptosis are still unclear.

Current drug development in cancer therapy is to induce mitogenic arrest via blocking diverse signal transduction pathways in cancer cells, and several chemotherapeutic agents such as paclitaxel and nocodazol that act against cancer cell cycle progression have been explored [20], [21]. It was indicated that mitotic arrest is a fundamental cause of cytotoxicity by these chemotherapeutic agents. Alternative expressions of cyclin-dependent kinases (CDKs) and cyclines drive progression of the cell cycle, and cyclinE/CDK2 for G_1_/S and cyclinB/CDK2 regulated by cdc25 for the G_2_/M transition were reported [22]. Clinical chemotherapeutic agents mainly cause cell cycle arrest at the G_2_/M phase and induce apoptosis in cancer cells. Activation of intracellular kinase cascades contributes to the proliferation and survival of cancer cells, and previous studies showed that activation of mitogen-activated protein kinases (MAPK), including extracellular signal-regulated kinase (ERK) and c-Jun N-terminal kinase (JNK) participates in apoptosis and cell cycle progression of cancer cells. Although induction of mitogenic arrest by EVO was reported, the role of MAPK activation in EVO-induced cell cycle arrest remains undefined.

In this study, we examined the mechanisms of EVO-inhibited viability and cell cycle progression of COLO205 and HT-29 colorectal carcinoma cells, and the structure-activity relationship (SAR) of EVO was analyzed. We found that EVO was able to reduce the viability of colorectal carcinoma cells via apoptosis induction, and G_2_/M arrest, which were independent of ROS production. Increased caspase-9 and -3 protein cleavage, and cyclin B1 and cdc25c proteins through induction of JNK protein phosphorylation by EVO were observed in colorectal carcinoma cells. Additionally, substitution at N14 of EVO is critical for apoptosis and G_2_/M arrest of colorectal carcinoma cells, and the intracellular pathway of apoptosis and G_2_/M arrest elicited by EVO was also investigated.

## Results

### EVO reduced the viability of colorectal carcinoma cells via apoptosis induction

**Figure 1:**
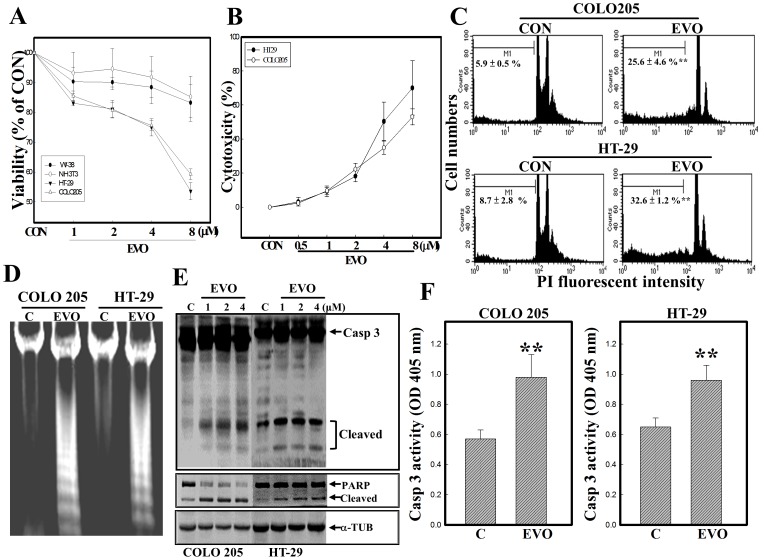
EVO reduction of viability of colorectal carcinoma COLO205 and HT-29 cells via apoptosis induction. (A) EVO reduction of cell viability of COLO205, HT-29, NIH3T3, and WI-38 cells by an MTT assay. These cells were treated with indicated concentrations (0.5, 1, 2, 4, and 8 µM) of EVO for 24 h, and cell viability was examined by an MTT assay. (B) EVO induction of lactate dehydrogenase (LDH) release by COLO205 and HT-29 cells according to an LDH release assay. As described in (A), the amount of LDH in the medium was examined by LDH kits. (C) Increased percentages of hypodiploid cells in EVO-treated COLO205 and HT-29 cell lines. Cells were treated with EVO (2 µM) for 24 h, and the percentage of hypodiploid cells was measured by flow cytometric analysis using PI staining. (D) EVO-induced loss of DNA integrity through increased DNA ladder formation. As described in (C), DNA integrity was analyzed by agarose electrophoresis. (E) Induction of caspase-3 (Casp 3) and poly(ADP ribose) polymerase (PARP) protein cleavage by EVO was detected in COLO205 and HT-29 cells by Western blotting using specific antibodies. (F) A significant increase in Casp 3 enzyme activity in EVO-treated colorectal carcinoma cells. As described in (C), activity of Casp 3 was measured by adding the Casp 3-specific colorimetric peptidyl substrate, Ac-DEVD-pNA. Each data point was calculated from three triplicate groups, and data are displayed as the mean ± S.D. ** p<0.01 denotes a significant difference compared to the control (C or CON) group.

In order to examine the effect of EVO on the viability of two colon carcinoma cells COLO205 and HT-29, MTT and LDH release assays were applied in the present study. In the study, NIH3T3 and WI-38 cells were used to test if the limited cytotoxicity of EVO against the viability of colon carcinoma cells. NIH3T3 cells were established cells from murine embryo with no tumor formation in mice, and WI-38 cells were isolated from normal embryonic lung tissue with a finite lifetime. As shown in Fig. 1A, concentration-dependent reductions in the viability of COLO205 and HT-29 cells were detected by the MTT assay, and EVO exhibits the more potent cytotoxicity again the viability of COLO205/HT-29 than NIH3T3/WI-38 cells. Data of the LDH release assay showed that EVO concentration-dependently increased LDH in the medium of COLO205 and HT29 cells (Fig. 1B). The ratio of apoptotic bodies indicated that increased apoptotic bodies were detected in EVO-treated COLO205 and HT-29 cells (Fig. 1C). Loss of DNA integrity with the appearance of DNA ladders was observed in EVO-treated COLO205 and HT-29 cells via DNA electrophoresis (Fig. 1D). Examination of apoptotic proteins including caspase-3 and PARP protein expressions showed that increased cleavage of caspase-3 and PARP proteins was detected in COLO205 and HT-29 cells under EVO stimulation (Fig. 1E). Additionally, caspase-3 activity induced by EVO was identified in COLO205 and HT-29 cells using the colorimetric peptidyl caspase-3 substrate, Ac-DEVD-pNA (Fig. 1F). These results supported the reduction in viability of colorectal carcinoma cells by EVO being mediated by induction of apoptosis.

### Mitochondrion-mediated apoptosis by EVO in colorectal carcinoma cells

**Figure 2:**
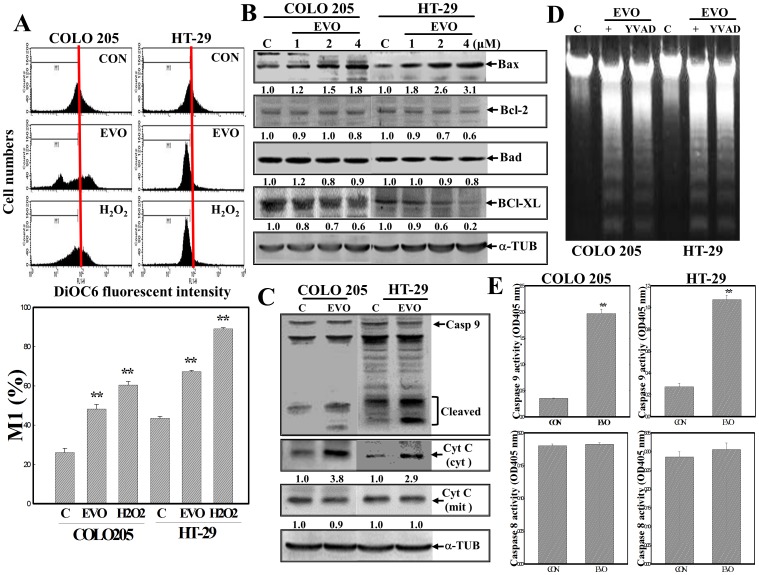
Disruption of the mitochondrial membrane potential (MMP) with increased Bax protein and cytosolic cytochrome (Cyt) c protein expressions, and caspase-9 (Casp 9) protein cleavage in EVO-treated COLO205 and HT-29 cells. (A) Loss of the MMP by EVO and H_2_O_2_ in COLO205 and HT-29 cells. Cells were treated with EVO (2 µM) or H_2_O_2_ (100 µM) for 12 h, and the MMP was detected by a flow cytometric analysis using DiOC6 as a fluorescent dye. (upper) A representative example of flow cytometric data is shown; (lower) quantification of the M_1_ ratio from three independent experiments is shown. (B) Alternative Bcl-2 family protein expression by EVO was detected by Western blotting using specific antibodies. Cells were treated with different concentrations of EVO for 24 h, and expressions of indicated proteins were detected by Western blotting. (C) EVO induction of Casp 9 protein cleavage and cytosolic Cyt c protein in COLO205 and HT-29 cells. As described in (C), expressions of Casp 9, cytosolic Cyt C, and mitochondrial Cyt c proteins were examined by Western blotting using specific antibodies. (D) The peptidyl Casp 9 inhibitor, Ac-YVAD-FMK (YVAD; 100 µM), inhibited EVO-induced DNA ladder formation by COLO205 and HT-29 cells. Cells were incubated with Ac-YVAD-FMK (100 µM) for 2 h followed by EVO (2 µM) treatment for 24 h, and DNA integrity was examined by agarose electrophoresis. (E) A significant increase in Casp 9, but not Casp 8, enzyme activity in EVO-treated colorectal carcinoma cells. As described in (C), activities of Casp 9 and 8 were respectively measured by adding the Casp 9-specific colorimetric peptidyl substrate, Ac-DEVD-pNA, or the Casp 8-specific colorimetric peptidyl substrate, Ac-IETD-pNA. Each data point was calculated from three triplicate groups, and data are displayed as the mean ± S.D. ** p<0.01 denotes a significant difference compared to the control (C or CON) group. The intensity of each band was examined by a densitometric analysis (Imag J), and expressed as multiples of the control.

We further examined the role of mitochondria in apoptosis induction by EVO in COLOL205 and HT-29 colorectal carcinoma cells. Data from the MMP analysis using a fluorescent mitochondria-binding dye (DiOC6) showed that EVO addition significantly reduced the MMPs in both cell lines. Reduction of the MMP by H2O2 was described as a positive control (Fig. 2A). Alternative expressions of pro- and antiapoptotic Bcl-2 family proteins appeared, in that an increase in the proapoptotic Bax protein and a decrease in the antiapoptotic protein Bcl-XL were observed by Western blotting using specific antibodies (Fig. 2B). Induction of cleavage of caspase-9 and Cyt c in cytosol was detected in EVO-treated COLO205 and HT-29 cells (Fig. 2C). Incubation of both cell lines with the peptidyl caspase-9 inhibitor, Ac-YVAD-FMK, inhibited EVO-induced DNA ladder formation (Fig. 2D). An increase in caspase-9, but not caspase-8, activity in EVO-treated COLO205 and HT-29 cells was observed using a specific peptidyl colorimetric substrate (Fig. 2E). These results indicated that disruption of the MMP contributed to EVO-induced apoptosis in colorectal carcinoma cells.

### ROS-independent apoptosis by EVO in colorectal carcinoma cells

**Figure 3:**
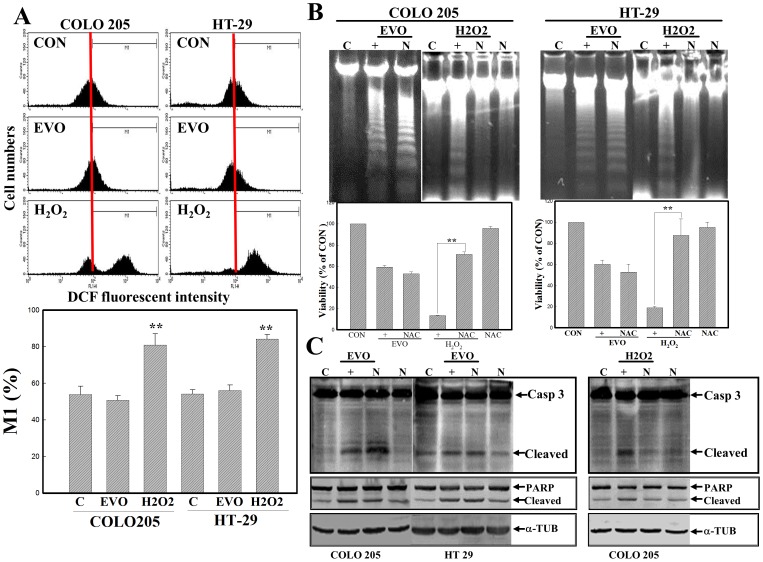
Role of reactive oxygen species (ROS) in EVO-induced apoptosis of colorectal carcinoma cells. (A) EVO shows no effect on intracellular peroxide production in COLO205 and HT-29 cells. Both cells were treated with EVO (2 µM) or H_2_O_2_ (100 µM) for 3 h followed by adding a fluorescent dye (DCHF-DA) to examine intracellular peroxide levels via a flow cytometric analysis. (upper) A representative example of flow cytometric data is shown; (lower) quantification of the M_1_ ratio from three independent experiments is shown. (B) N-Acetyl-L-cysteine (NAC; N; 20 mM) protected cells from H_2_O_2_-induced cell death and DNA ladder formation, but had no effect on EVO-induced apoptosis. Both cells were treated with NAC (20 mM) for 30 min followed by EVO (2 µM) or H_2_O_2_ (100 µM) treatment for 24 h. DNA integrity (upper panel) and the viability (lower panel) of cells under different treatments are examined by agarose electrophoresis and MTT assay, respectively. (C) NAC inhibited H_2_O_2_-induced caspase (Casp) 3 and poly(ADP ribose) polymerase (PARP) protein cleavage, but did not affect EVO-induced events in COLO205 and HT-29 cells. As described in (B), the indicated protein expression was examined by Western blotting using specific antibodies. Each data point was calculated from three triplicate groups, and data are displayed as the mean ± S.D. **p<0.01 denotes a significant difference compared to the control (C) in (A) or between indicated groups (B).

We further examined the role of ROS in EVO-induced apoptosis in COLO205 and HT-29 cells. Intracellular peroxide levels were detected by a flow cytometric analysis using DCHF-DA as a fluorescent dye. As illustrated in Fig. 3A, the addition of H_2_O_2_ induced intracellular peroxide production in COLO205 and HT-29 cells, but no effect on peroxide production in either cell line by EVO was observed. Additionally, H2O2-induced DNA ladders and cytotoxicity were detected in COLO205 and HT-29 cells, and those were abolished by adding the antioxidant, NAC, via agarose electrophoresis and an MTT assay, respectively. However, NAC addition was unable to reduce EVO-induced cell death via MTT assay and DNA ladders via agarose electrophoresis in either cell line (Fig. 3B). Increases in cleaved caspase-3 and PARP proteins with H_2_O_2_ treatment were detected by Western blotting using specific antibodies, and they were inhibited by the addition of NAC (Fig. 3C). No alteration in the expressions of cleaved caspase-3 or PARP protein was observed in EVO- and EVO+NAC-treated cells. This indicates that EVO-induced apoptosis might not be mediated in an ROS-dependent manner in colorectal carcinoma cells.

### Activation of JNK is involved in EVO-induced apoptosis of colorectal carcinoma cells

**Figure 4:**
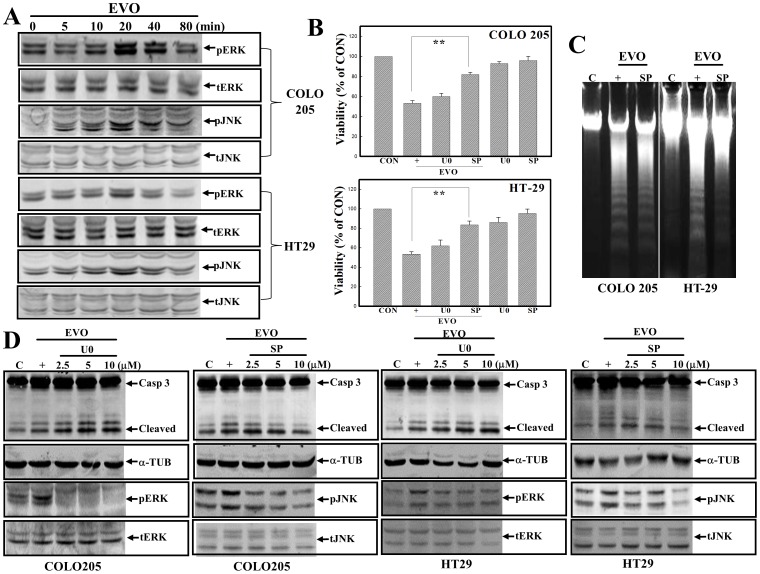
c-Jun N-terminal kinase (JNK) activation participates in EVO-induced apoptosis of COLO205 and HT-29 cells. (A) Induction of extracellular signal-regulated kinase (ERK) and JNK protein phosphorylation by EVO in colorectal carcinoma cells. Both cell lines were treated with EVO (2 µM) for different times, and expressions of phosphorylated (p)ERK/(p)JNK and total (t)ERK/(t)JNK were detected by Western blotting using specific antibodies. (B) The JNK inhibitor, SP600125 (SP; 20 µM), but not the ERK inhibitor, U0126 (U0; 20 µM), protected COLO205 and HT-29 cells from EVO-induced cytotoxicity according to an MTT assay. (C) SP600125 attenuates EVO-induced DNA ladder formation in colorectal carcinoma cells. Cells were treated with SP600125 (10 µM) for 30 min followed by EVO stimulation for an additional 24 h, and DNA integrity was examined by agarose electrophoresis. (D) SP600125 inhibited EVO-induced JNK protein phosphorylation and caspase (Casp) 3/poly(ADP ribose) polymerase (PARP) protein cleavage; however, U0126 inhibited EVO-induced ERK protein phosphorylation without affecting EVO-induced Casp 3/PARP protein cleavage in both cell lines. Both cell lines were treated with different concentrations of SP600125 or U0126 for 30 min followed by EVO stimulation for 30 min (for ERK and JNK protein expressions) or 24 h (for Casp 3 and PARP protein expressions) via Western blotting. Each data point was calculated from three triplicate groups, and data are displayed as the mean ± S.D. **p<0.01 denotes a significant difference compared between indicated groups.

The roles of MAPK activation were reported, and we investigated if EVO-induced apoptosis occurred through altered MAPK activation in colorectal carcinoma cells. As shown in Fig. 4A, increased ERK and JNK protein phosphorylation was detected in COLO205 and HT-29 cells under EVO stimulation. Incubation of both cell lines with the ERK inhibitor, U0126, or the JNK inhibitor, SP600125, followed by EVO stimulation was applied to examine the roles of ERK and JNK activation in EVO-induced apoptosis. Data of the MTT assay showed that SP600125, but not U0126, addition protected both colorectal carcinoma cell lines from EVO-induced cell death (Fig. 4B). Examination of DNA integrity showed that SP600125 addition attenuated EVO-induced DNA ladder formation in COLO205 and HT-29 cells (Fig. 4C). Data of Western blotting showed that U0126 inhibited EVO-induced pERK protein expression, but did not affect EVO-induced cleavages of caspase-3 protein in COLO205 and HT-29 cells. In the same part of the experiment, SP600125 exhibited an inhibitory effect on EVO-induced pJNK protein in accordance with the decrease in cleavage of the caspase-3 protein in both cell lines. No changes in the expressions of α-tubulin, tERK, or tJNK were described as internal controls (Fig. 4D).

### Induction of G2/M arrest and cyclin B1/cdc25c protein expression by EVO were identified in COLO205 and HT-29 cells, and these were blocked by adding the JNK inhibitor, SP600125

**Figure 5:**
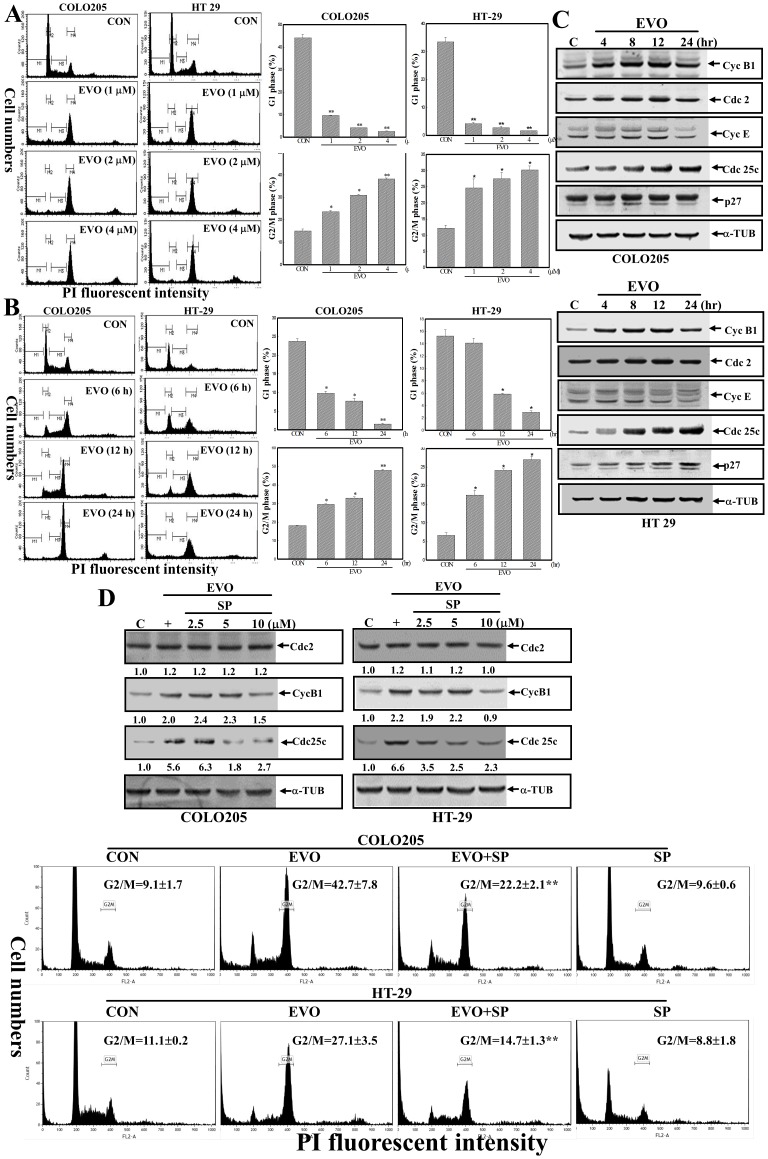
Induction of G_2_/M arrest and cyclin B1/cdc 25c protein expressions by EVO in COLO205 and HT-29 cells was significantly inhibited by adding the c-Jun N-terminal kinase (JNK) inhibitor, SP600125. (A) Concentration-dependent increases in the G_2_/M ratio and decreases in the G_1_ ratio in EVO-treated COLO205 and HT-29 cells. Both cell lines were treated with different concentrations (1, 2, and 4 µM) of EVO for 24 h, and ratios of cells in the G_1_ and G_2_/M phases were measured by a flow cytometric analysis via PI staining. (B) Time-dependent increases in the G_2_/M ratio and decreases in the G_1_ ratio were detected in EVO-treated colorectal carcinoma cells. Cells were treated with EVO (2 µM) for different times (6, 12, and 24 h), and ratios of cells in the G_1_ and G_2_/M phases were measured by a flow cytometric analysis via PI staining. (Left panel) A representative example of flow cytometric data is shown. (Right panel) Data of the G_1_ and G_2_/M ratios from three independent experiments are presented. (C) Alternative expressions of cell cycle regulatory proteins including cyc B1, cdc 2, cyc E, cdc 25c, p27, and α-tubulin in colorectal carcinoma cells under EVO stimulation. Cells were treated with EVO (2 µM) for different times (4, 8, 12, and 24 h), and expressions of the indicated proteins were examined by Western blotting using specific antibodies. (D) The JNK inhibitor, SP600125 (SP), inhibited EVO-induced cdc25c and cyc B1 protein expressions accompanied by decreases in the G_2_/M ratio in COLO205 and HT-29 cells. (Upper panel) Cells were treated with different concentrations of SP600125 for 30 min followed by EVO (2 µM) stimulation for 24 h, and expression of the indicated protein was examined by Western blotting. (Lower panel) Cells were treated with SP600125 (10 µM) for 30 min followed by EVO (2 µM) treatment for 24 h, and cell cycle progression was analyzed by flow cytometry via PI staining. The intensity of each band was examined by a densitometric analysis (Imag J), and expressed as multiples of the control. Each data point was calculated from three triplicate groups, and data are displayed as the mean ± S.D. **p<0.01 denotes a significant difference compared to the control (CON).

Cell cycle progression was analyzed by flow cytometry using PI as a fluorescent dye. As illustrated in Fig. 5A, an increase in the G2/M ratio and a decrease in the G1 ratio were detected in EVO-treated COLO205 and HT-29 cells in concentration-dependent manners. Similarly, the EVO-induced G2/M ratio and EVO-inhibited G1 ratio were observed in both COLO205 and HT-29 cells in time-dependent manners (Fig. 5B). Altered expressions of cell cycle-regulatory proteins including cyc B1, cdc 2, cyc E, cdc 25c, and p27 were detected by Western blotting using specific antibodies, and data shown in Fig. 5C reveal that time-dependent increases in cyc B1 and cdc 25c, but not the others, were detected in both EVO-treated cell lines. Increases in cyc B1 and cdc 25c proteins by EVO were reduced with a decrease in EVO-induced G2/M by adding the JNK inhibitor, SP (Fig. 5D). These results indicated that JNK activation contributes to EVO-induced apoptosis and G2/M arrest of colorectal carcinoma cells.

### Structure-activity analysis of effects of EVO-related compounds on the viability of colorectal carcinoma cells

**Figure 6:**
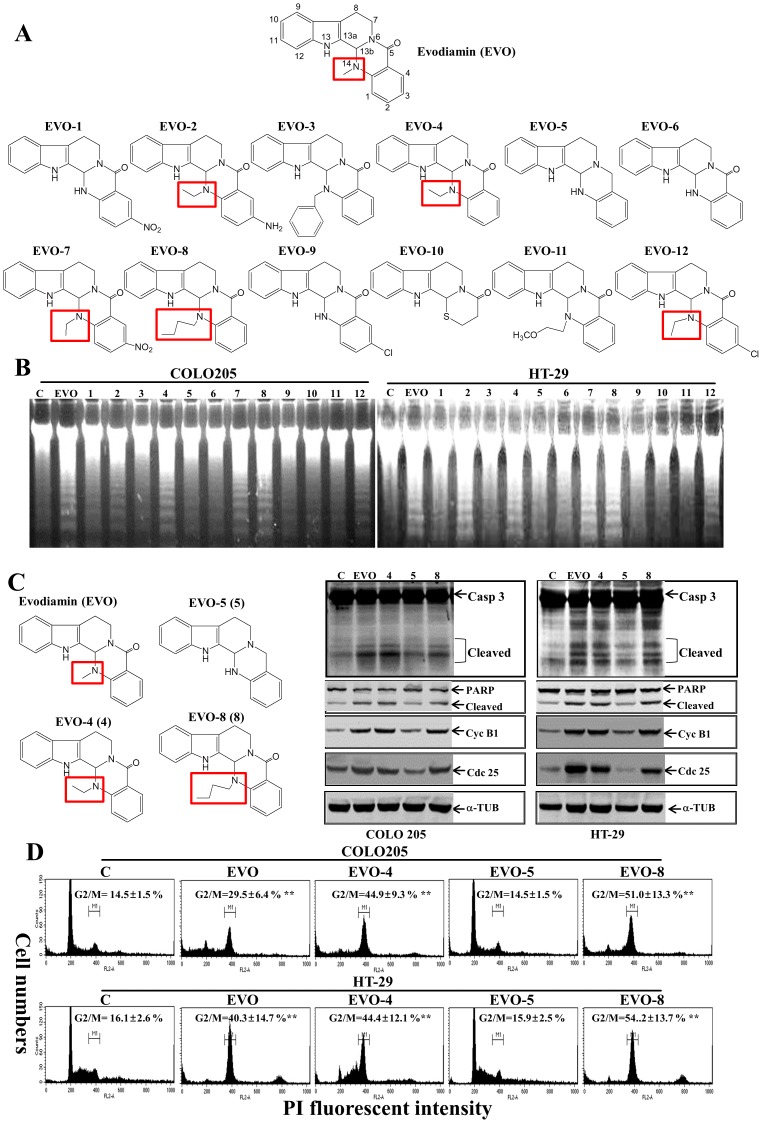
Structure-activity relationship of EVO and related chemicals on apoptosis and G_2_/M arrest elicited by EVO in colorectal carcinoma cells. (A) The chemical structures of EVO and structurally related chemicals (EVO-1∼12) are depicted. (B) Differential apoptotic effects elicited by EVOs in colorectal carcinoma cells. Cells were treated with the indicated EVOs (2 µM) for 24 h, and DNA integrity was analyzed by agarose electrophoresis. (C) Four EVOs with different substitutions at the position 14 of quinazolin showed differential effects on caspase (Casp) 3/poly(ADP ribose) polymerase (PARP) protein cleavage and cycB1/cdc 25c protein expressions in colorectal carcinoma cells. Cells were treated with the indicated chemicals (2 µM) for 24 h, and expressions of Casp 3/PARP, cycB1/cdc 25c, and α-tubulin (TUB) were detected by Western blotting using specific antibodies. (D) EVO, EVO4 (4), and EVO-8 (8), but not EVO-5 (5), increased the G_2_/M ratio of COLO205 and HT-29 cells. As described in (C), the G_2_/M ratio of COLO205 and HT-29 cells under different treatments was examined by flow cytometric analysis via PI staining. Each data point was calculated from three triplicate groups, and data are displayed as the mean ± S.D. **p<0.01 denotes a significant difference compared to the control (CON).

EVO and 12 EVO-related compounds were applied to examine their effects on apoptosis induction of COLO205 and HT-29 cells. Structures of these chemicals are depicted in Fig. 6A, and different substitutions at position 14 of EVO are marked. Analysis of the DNA integrity in COLO205 and HT-29 cells under treatment with the indicated EVO-related chemicals showed that EVO, EVO-2, -4, -7, -8, and -12 possessed the ability to induce DNA ladder formation in both cell lines, whereas EVO-1, -3, -5, -6, -9, -10, and -11 did not (Fig. 6B). This implies that adding an alkyl group, such as a methyl or butyl, at position 14 of quinazolin is critical to apoptosis induction by EVO. Furthermore, four compounds (i.e., EVO, -4, -5, and -8) were selected for a mechanism study, and these EVOs contained the same structure except for a methyl of EVO, an ethyl of EVO-4, a hydrogen of EVO-5, and a butyl of EVO-8 at position 14. Western blotting data showed that EVO, EVO-4, and EVO-8 induced cleavage of caspase-3 and PARP proteins with increased cyc B1 and cdc25c protein levels in COLO205 and HT-29 cells; however, EVO-5 did not (Fig. 6C). Analysis of the G2/M ratio in EVO-treated COLO205 and HT-29 cells showed that significant increases in the G2/M ratio were detected in EVO, EVO-4, and EVO-8-treated cells, but those were not observed in EVO-5-treated cells (Fig. 6D).

## Discussion

Cell cycle checkpoints play important roles in coordination of cell cycle transitions in eukaryotic cells, and abnormal regulation of cell cycle checkpoints are frequently occurred in tumor cells. The function of arrest at cell cycle checkpoints is for DNA repair when cellular damage occurred, and the cell cycle arrest signaling might activate apoptotic pathways, leading to cell death when cellular damage is irreparable. According to flow cytometric analysis, EVO-induced G_2_/M arrest in COLO205 and HT-29 cells occurred at an early time point (Fig. 5B; 6 h) with a subsequent increase in apoptotic ratio at later times (12 and 24 h). It indicates G_2_/M arrest resulted in the inhibition of cell proliferation leading to apoptosis by EVO in COLO205 and HT-29 cells. There is growing evidence indicating that inappropriate activation of cdc25c and cyclin B1 has an important role in antitubulin agent induced mitotic arrest and apoptosis. Increases in the G2/M ratio and cyclin B1/cdc 25c protein expression by EVO were observed in COLO205 and HT-29 cells, and the JNK inhibitor, SP600125, but not the ERK inhibitor, U0126, inhibited EVO-induced JNK protein phosphorylation with suppression of EVO-induced apoptotic events and G2/M arrest in COLO205 and HT-29 cells. Taken together, the molecular mechanism for EVO's induction of apoptosis and G2/M arrest in a JNK-mediated manner in colorectal carcinoma cells was demonstrated in the present study.

Caspase-3 is a critical executioner of apoptosis through cleaving several essential cellular proteins such as PARP and D4-GDI. Induction of apoptosis through activation of caspase activity by EVO was reported in several previous studies. Huang et al. (2004) and Zhang et al. (2010) reported that EVO induced apoptosis and cleavage of caspases in human leukemic T lymphocytes and colon LOVO cells [15], [23]. Zhang et al. (2013) reported that EVO induced caspase-dependent and -independent apoptosis in human U937 leukemia cells [9]. Wang et al. (2013) reported that EVO inhibited the proliferation and induced cleavage of caspase-7 and PARP in breast carcinoma cells [24]. Our investigations revealed that EVO has the ability to increase caspase-3 activity and expressions of cleaved caspase-3 and PARP proteins, accompanied by apoptosis induction in COLO205 and HT-29 cells. These findings show that activation of the caspase cascade contributes to EVO-induced apoptosis in colon carcinoma cells.

A low level of reactive oxygen species (ROS) is important for cellular function and survival signaling, while excessive ROS-elicited oxidative stress leads to cell death via apoptosis induction. Accumulated evidence indicates that chemotherapeutic agents can induce apoptosis through ROS production in various cancer cells. Yang et al. (2011) reported that increased ROS production by gelomulide K potentiates the lethality of breast carcinoma cells [25]. Our previous publications supported an involvement of ROS production in apoptosis of cancer cells [14], [26], [27]. Imatinib mesylate, gossypol, vitamin K3, and flavonoids induced apoptosis in several cell lines including melanoma, leukemia, glioma, and colorectal carcinoma cells through elevating ROS production. However, ROS-independent apoptosis by chemical stimulation was also reported [28], [29]. Although apoptosis induced by EVO was reported, the roles of ROS are still undefined. In the present study, NAC inhibited H_2_O_2_-induced DNA ladder formation and caspase-3/PARP protein cleavage, but was unable to block EVO-induced apoptosis. Data of DCHF-DA staining indicated that no alteration in intracellular peroxide levels by EVO was observed in COLO205 or HT-29 cells. These results suggested that ROS might not be involved in EVO-induced apoptosis of colorectal carcinoma cells. In contrast to those results, EVO elevation of ROS and NAC inhibition of EVO-induced apoptosis in human cervical carcinoma HeLa cells were observed. Lower concentrations (1∼4 µM) of EVO in colon carcinoma cells in the present study and a higher concentration (21 µM) in cervical carcinoma cells in the previous study are possibly why ROS played differential roles in EVO-induced apoptosis. Additionally, Bcl-2 family proteins participate in maintenance of MMP regulation of the release of mitochondrial Cyt c to the cytosol and activation of caspase-9 activity which contribute to apoptosis of cancer cells. A significant increase in the proapoptotic Bax protein with decreases in antiapoptotic Bcl-2/Bcl-xL proteins was identified in both COLO205 and HT-29 cells under EVO stimulation. Accordingly, loss of the MMP with the occurrence of caspase-9 protein cleavage and release of Cyt C from mitochondria to the cytosol was observed in EVO-treated cells. Mitochondrion-dependent apoptosis by EVO was indicated to occur in colorectal carcinoma cells.

MAPK is implicated in regulating survival and cell death responses of tumor cells, and several studies reported the involvement of MAPK in cancer deregulation; however the precise mechanisms of MAPK in apoptosis and cell cycle progression of cancer cells remain elusive. Du et al. (2013) reported that EVO-induced apoptosis was enhanced by its combination with the ERK inhibitor, PD98059, or the p38 MAPK inhibitor, SB203580 [30]. The relationship of MAPK to EVO-induced apoptosis and cell cycle arrest is still unclear. Data of the present study indicated that induction of ERK and JNK protein phosphorylation by EVO was detected in COLO205 and HT-29 cells, and EVO-induced apoptotic events, including DNA ladder formation and caspase-3 protein cleavage, were inhibited by adding the JNK inhibitor, SP600125, but not the ERK inhibitor, U0126. Additionally, control of cell cycle progression in cancer cells is regarded as an effective strategy for inhibiting tumor cell proliferation. Previous studies reported that EVO inhibited the proliferation of various cancer cells that were arrested at the G2/M or S phase [23], [31], but the mechanism for mitogenic arrest by EVO is still poorly understood. In the present study, an increased G2/M ratio by EVO with induction of cyclinB1 and cdc25c protein expressions was detected in COLO205 and HT-29 cells. Addition of the JNK inhibitor, SP600125, decreased EVO-induced G2/M arrest and cyclinB1/cdc25c protein expression in both colon carcinoma cell lines. The promoters of *cyclin B* and *CDC25C* conserved cell cycle-dependent element (CDE), cell cycle genes homology region (CHR) sites, and CCAAT-boxes. Several factors such as E2F, CDF-1, and CBP have been reported to bind with CHR/CDE in *cyclin B* and *CDC25C* promoters [32]. Muller et al (2012) found that CHR is a central element in transcriptional regulation of *cyclin B* by the DREAM and MMB complexes [33]. Chae et al (2011) found a transcriptional factor NF-Y binds to CCAAT in the promoters of cell cycle G2 regulators such as *cyclin B* and *CDC25C* [34]. Seo et al (2008) indicated that phosphorylated c-Myc bound to the promoter of cyclin B1, resulting in increased cyclin B1 promoter activity [35]. Inhibition of JNK protein phosphorylation reduces cdc25c/cyclinB1 protein expression in EVO-treated COLO 205 and HT-29 cells, however the mechanism of JNK inhibition leading to reduce EVO-induced cdc25c/cyclinB1 protein expression is still unclear. Contribution of JNK to transcriptional regulations of *cyclin B1* and *CDC25C* gene via modulating the binding of transcriptional factors to their promoters needs to be further investigated.

In order to estimate the structures that contribute to the apoptosis and G2/M arrest induced by EVO in colorectal carcinoma cells, the effects of compounds (EVO-1∼12) possessing structures similar to that of EVO on apoptosis and cell cycle progression of both colon cancer COLO205 and HT-29 cell lines were examined. As shown in Fig. 6, EVO-2, -4, -7, -8, and -12 containing an alkyl group such as ethyl or butyl at position 14 compared to the methyl group of EVO induced significant apoptosis in COLO205 and HT-29 cells. Furthermore, EVO and its structurally related compounds including EVO-4, -5, and -8 were used to study the effects on caspase-3, PARP, cyclinB1, and cdc25c protein expressions with cell cycle progression in both colorectal carcinoma cell lines. EVO, EVO-4, -5, and -8 share the same chemical structure except for different substitutions including a methyl of EVO, an ethyl of EVO-4, a hydrogen of EVO-5, and a butyl of EVO-8 at position 14. Our results showed that EVO, EVO-5, and EVO-8, but not EVO-4, significantly induced G2/M arrest with increased cyclin B1/cad25c protein expressions and caspase-3/PARP protein cleavage in both colon carcinoma cell lines. Ogasawara et al. (2002) also indicated the role of a methyl group at position 14 for EVO in inhibiting invasion by Lewis lung cancer and melanoma cells [11]. The critical roles of alkyl substitutions such as methyl and butyl at position 14 for apoptosis and G2/M arrest by EVO against colorectal carcinoma cells were demonstrated.

In conclusion, we showed in the present study that EVO possesses antitumor activities including apoptosis and G2/M arrest against the viability of colorectal carcinoma cells. EVO induced disruption of the MMP, which was accompanied by activation of caspases-3/9, and increases in cyclin B1/cdc25c protein expressions in COLO205 and HT-29 cells. Activation of JNK by EVO was detected, and EVO-induced apoptotic and G2/M arrest were blocked by the JNK inhibitor, SP600125, indicating the critical role of JNK activation in the anti-colorectal carcinoma activity of EVO. Furthermore, a structure-activity study showed that methyl at position 14 is important for EVO's action against the viability of colon cancer cells. Further studies will investigate whether these effects of EVO can be extended to colon cancer cells in vivo, especially chemotherapy-resistant colon cancer cells.

## Methods

### Cell culture

COLO205, HT-29, NIH3T3, and WI-38 cells were obtained from the American Type Culture Collection (Manassas, VA, USA). COLO205/HT-29 colon carcinoma cells in RPMI 1640, WI-38 in MEM containing 10% heat-inactivated fetal bovine serum (FBS; Gibco/BRL, Grand Island, NY, USA), and NIH3T3 in DMEM containing 10% heat-inactivated calf serum (CS; Gibco/BRL, Grand Island, NY, USA), supplemented with antibiotics (100 U/mL penicillin A and 100 U/mL streptomycin) were maintained in a 37 °C humidified incubator containing 5% CO_2_.

### Agents

The chemical reagents of EVO, N-acetyl-l-cysteine (NAC), SP600125, U0126, BCIP, 3-(4,5,-dimethylthiazol)-2-yl-2,5-diphenyltetrazolium bromide (MTT), and NBT were obtained from Sigma Chemical (St. Louis, MO, USA). Antibodies of α-tubulin, poly(ADP ribose) polymerase (PARP), caspase-3, caspase-9, Bcl-2, and Bax were obtained from Santa Cruz Biotechnology (Santa Cruz, CA, USA). Antibodies of total (t) and phosphorylated (p) MAPKs (tERK/pERK and tJNK/pJNK), and cyclinB1/cdc25c proteins were obtained from Cell Signaling Technology (Danvers, MA, USA). The colorigenic synthetic peptide substrates, Ac-DEVD-pNA (a caspase-3 substrate), Ac-YVAD-pNA (a caspase-9 substrate), and Ac-IETD-pNA (a caspase-8 substrate) were purchased from Calbiochem. Other chemicals not mentioned above were obtained from Sigma Chemical.

### Synthesis of structure-related chemicals of EVO

The synthesis of EVO-related compounds were based on the coupling of 3,4-dihydro-β-carboline with substituted N-alkyl isatoic anhydride in pyridine. 3,4-dihydro-β-carboline was prepared by reacting tryptamine with ethyl formate and followed by intramolecular ring closure in the presence of POCl_3_. In the presence of NaH and DMF, Isatoic anhydride was alkylated with alkyl halide such as iodomethane, iodoethane, iodoprpopane, 2-methoxy ethyl chloride to afford N-alkyl isatoic anhydride analogues. The purities of them were more than 95% when analyzed by HPLC (Fig. S1).

### MTT (3-(4,5,-dimethylthiazol)-2-yl-2,5-diphenyltetrazolium bromide) assay

Cell viability was assessed by MTT staining as described previously [13]. Briefly, cells were plated at a density of 10^5^ cells/well into 24-well plates. At the end of treatment, the supernatant was removed, and 30 µl of the tetrazolium compound, MTT, and 270 ml of fresh RPMI medium were added. After incubation for 4 h at 37°C, 200 µl of 0.1 N HCl in 2-propanol was placed in each well to dissolve the tetrazolium crystals. Finally, the absorbance at a wavelength of 600 nm was recorded using an enzyme-linked immunosorbent assay (ELISA) plate reader.

### Lactate dehydrogenase (LDH) release assay

The percentage of LDH release was expressed as the production of LDH released into the medium compared to the total amount of LDH present in cells treated with 2% Triton X-100. The activity was monitored by the oxidation of the reduced form of NADH at 530 nm by an LDH assay kit (Roche, Indianapolis, IN, USA). Cytotoxicity was determined by the equation: [(OD530 of the treated group – OD530 of the control group)/(OD530 of the Triton X-100-treated group – OD530 of the control group)] × 100%.

### Western blotting

Total cellular extracts (30 µg) were prepared and separated on 8% sodium dodecylsulfate (SDS)-polyacrylamide mini gels for PARP detection and 12% SDS-polyacrylamide minigels for caspase-3, caspase-9, the Bcl-2 family, tERK, pERK and α-tubulin detection, and transferred to Immobilon polyvinylidene difluoride membranes (Millipore, Bedford, MA, USA). Membranes were incubated at 4 °C with 1% bovine serum albumin (BSA) and then incubated with the indicated antibodies for a further 3 h at room temperature followed by incubation with an alkaline phosphatase-conjugated immunoglobulin G (IgG) antibody for 1 h. Proteins were visualized by incubating with the colorimetric substrates, nitroblue tetrazolium (NBT) and 5-bromo-4-chloro-3-indolyl phosphate (BCIP).

### Measurement of reactive oxygen species (ROS) generation by colonic carcinoma cells

Intracellular production of ROS by colonic carcinoma cells under different treatments was measured by oxidation of DCFH-DA to DCF. DCFH-DA is a non-polar compound that readily diffuses into cells, where it is hydrolyzed to the non-fluorescent polar derivative, DCFH, and is thereby trapped within cells. When DCFH-DA is oxidized, it turns into the highly fluorescent DCF. After treatment, cells were incubated in the dark for 10 min at 37 °C with 50 µM DCFH-DA, then 10^4^ cells were acquired per sample, the fluorescence of cells was analyzed using a FACScan (Becton Dickinson, Sunnyvale, CA, USA) flow cytometer with excitation at 488 nm and emission at 530 nm.

### DNA fragmentation assay

Trypsinized cells were washed with ice-cold phosphate-buffered saline (PBS) and fixed in 70% ethanol at −20 °C for at least 1 h. After fixation, cells were washed twice with PBS and incubated in 1 ml of 0.5% Triton X-100/PBS at 37 °C for 30 min containing 1 mg/ml of RNase A, followed by staining with 1 ml of 50 µg/ml propidium iodide (PI) for 10 min. Fluorescence emitted from the PI-DNA complex was quantitated after excitation of the fluorescent dye by FACScan flow cytometry (Becton Dickenson, San Jose, CA, USA). Ratios of cells at the G_2_/M and sub-G_1_ phases were measured, and expressed as percentages (%) of total counts.

### Measurement of the mitochondrial membrane potential (MMP)

After different treatments, cells were incubated with 40 nM DiOC6(3) for 15 min at 37 °C, then washed with ice-cold PBS, and collected by centrifugation at 500×*g* for 10 min. Collected cells were resuspended in 500 ml of PBS containing 40 nM DiOC6(3). Fluorescence intensities of DiOC6(3) were analyzed on a flow cytometer (FACScan, Becton Dickinson) with excitation and emission settings of 484 and 500 nm, respectively.

### Analysis of caspase-3, -8, and -9 activities

Ac-DEVD-pNA was used as a colorimetric protease substrate to detect caspase-3 activity. After different treatments, cells were collected and washed three times with PBS and resuspended in 50 mM Tris–HCl (pH 7.4), 1 mM EDTA, and 10 mM EGTA. Cell lysates were clarified by centrifugation at 15,000 rpm for 3 min, and clear lysates containing 200 µg of protein were incubated with 100 µM of the indicated specific colorimetric substrates at 37 °C for 1 h. Alternative activities of indicated caspase-3, -8, and -9 enzymes were described as the cleavage of colorimetric substrate by measuring the absorbance at 405 nm.

### Detection of cell cycle progression and hypodiploid cells by EVO in colon carcinoma cells

Cells were plated in 24 well plates in duplicate, then incubated for 24 h. Media were removed and different treatments were added to each well. Cells were treated for 12 h and the supernatant and cells were then harvested by exposing the cells to 0.25%, Trypsin-EDTA solution for 10 min, then centrifuged and washed in phosphate buffered saline (PBS), fixed in 3 mL ice-cold 100% ethanol. All samples were incubated for 30 min at room temperature in the dark. Cell cycle distribution and hypodiploid cells were determined using FACSan Flow Cytometer (FACScan, Becton Dickinson).

### Statistical analysis

Values are expressed as the mean±standard error (SE) of triplicate experiments. The significance of the difference from the respective controls for each experimental was assayed using a one-way analysis of variance (ANOVA) with a post-hoc Bonferroni analysis when applicable, and *p* values of <0.05 or <0.01 were considered statistically significant.

## Supporting Information

Figure S1 (PPTX)
