# Supplementary figures and images for: Activation of JNK Contributes to Evodiamine-Induced Apoptosis and G2/M Arrest in Human Colorectal Carcinoma Cells: A Structure-Activity Study of Evodiamine
Source: PLoS One. 2014 Jun 24;9(6):e99729. doi: 10.1371/journal.pone.0099729 (PMC4069003; doi:10.1371/journal.pone.0099729)

## Slide 1
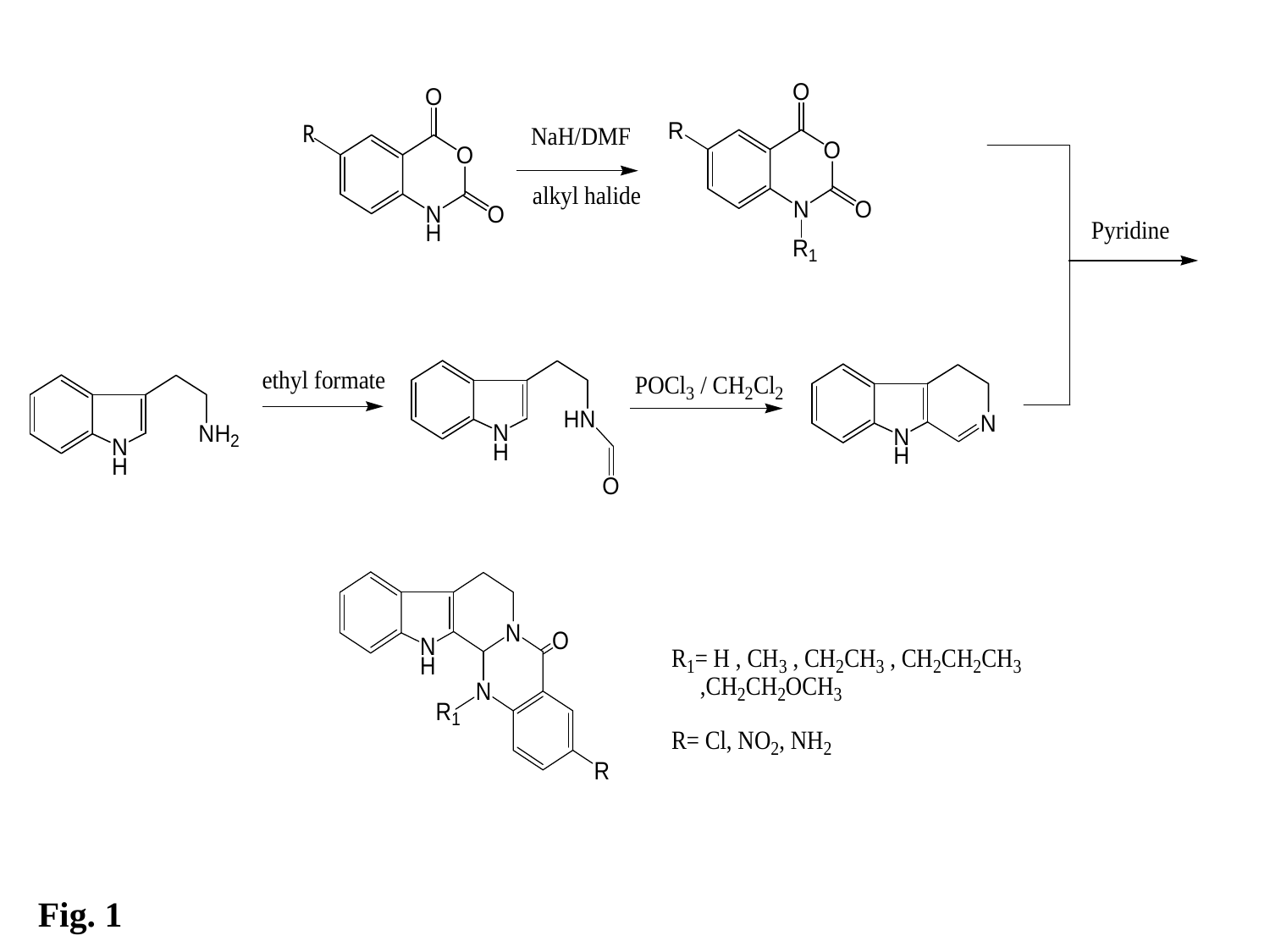

Fig. 1

Supplement: Figure S1 — (PPTX) [file pone.0099729.s001.pptx]
